# Author Index for Volume 101

**DOI:** 10.1038/sj.bjc.6605475

**Published:** 2009-12-08

Aapro, M 1961

Aaronson, NK 1505

Abdoun, M 1207

Abe, M 983

Abhyankar, A 1596

Absolom, K 561, 568

Aburatani, H 145, 1220

Adamcová, M 792

Adamski, JK 55

Adeney, EM 498

Adolfsson, J 902

Agelaki, S 589

Ahlgren, G 1233

Ahlskog, JKJ 645

Ahmed, HU 19, 2056, 2059

Ahn, J 522

Aigner, M 457

Aittomäki, K 1456

Akahori, T 1709

Akrivos, T 1059

Akslen, LA 1282

Alam, F 124

Ala-opas, M 843

Al-Attar, A 1321

Albanes, D 522

Albano, RM 782

Albrecht, S 722

Alcaraz, A 1248

Alfred Yung, WK 615

Ali, A 951

Alì, G 1869

Allasia, C 48

Allavena, A 2048

Allen, C 19

Allen, KE 1676

Allen, NE 192

Allory, Y 951

Alonso-González, C 1613

Alston, RD 1939

Alvarado-Ibarra, M 860

Alvarez-García, V 1613

Amant, F 244

Amaral, AT 80

Ambroisine, L 1137

Ammar, A 645

Amorim, J 973

Anders, M 1574

Anderson, AR 1805

Anderson, C 882

Andersson, S 511

Andersson, S-O 1932

Andersson, Y 1307

Ando, M 1529

Andrac, L 48

Andrade, R 64

Andreopoulou, E 1813

Andrulis, IL 1456, 2048

Anjomshoaa, A 822

Anninga, JK 1909, 2068

Anseline, P 759

Anthoney, A 621

Anton-Culver, H 1805

Antonini, NF 357

Antoniou, AC 1456, 2048

Anttinen, J 1005

Aogi, K 1676

Aparicio, LA 1248

Apostolaki, S 589

Apostolidou, S 160

Appleby, PN 192

Aquilani, L 1066

Arai, T 1676

Araki, K 116, 492

Araki, T 1648

Araud, L 350

Ardnor, B 91

Areal, J 1248

Aréchaga, J 64

Arenas, RB 1683

Arlucea, J 64

Armaou, S 32

Armitage, JN 19

Armstrong, JL 1448

Arnold, D 1241

Arnold, N 1456, 2048

Arnould, L 1357

Arora, RS 1939

Arriagada, R 902

Artale, S 1261

Arvanitis, C 38

Arver, B 2048

Asaga, T 598, 1031

Ascoli, V 1085

Ashcroft, F 1145

Ashraf, SQ 1758

Ashworth, A 973

Askmalm, MS 2048

Atagi, S 1537

Athanasiou, A 32

Athanasopoulos, PS 32

Atkins, J 1543

Atkinson, J 722

Attard, G 1137

Aubert, S 637

Austin, SC 483

Auvinen, A 843

Ay, A-S 673

Ayad, M 1207

Azodi, M 335

Baas-Vrancken Peeters, M-J 1505

Baba, T 269

Baborie, A 124

Bachet, J-B 7

Bäcklund, ML 1919

Bafna, S 1155

Bågeman, E 1817

Bahar, G 1194

Bahar, R 1329

Bai, J 327

Baka, S 621

Baker, M 1839

Bakkali, H 1207

Balasubramanian, SP 666, 1692

Ball, S 774

Ban, N 363

Banito, A 1782

Banks, RE 1175

Banu, N 1124

Barabási, A-L 749

Barathova, M 1290

Bardella, L 782

Bardelli, A 1261

Barile, M 2048

Barjhoux, L 2048

Barker, J 957

Barnett-Griness, O 2048

Barone, C 1162, 1261

Barrett, T 1522

Bartlett, JB 803

Barwell, JG 403

Basil, J 2048

Basolo, F 715

Bastian, BC 813

Bataille, R 166

Bates, DO 1183

Bates, T 395, 1032

Batra, SK 1155

Bauer, J 813

Baumforth, KRN 1393

Baxter, D 1502

Bearz, A 734

Beauchet, A 7

Becquart, D 232

Bedadra, W 1207

Beer, GM 605

Beesley, J 1456, 1805, 2048

Behl, S 1846

Behrens, R 1846

Behrmann, C 1241

Bekkers, RLM 27

Belien, JAM 1011

Bellet, D 473

Bellocco, R 1932

Bellone, S 335

Ben-Ayed, F 1207

Ben-Ayoub, W 1207

Bencardino, K 715

Benchimol, S 1555

Benider, A 1207

Benítez, J 1456, 1469, 2048

Bennicelli, E 219

Berchuck, A 269, 1461, 1805

Bergh, J 902

Bergmann, F 457

Bergstrom, P 1995

Bermejo, JL 935

Bernaldez-Rios, R 860

Bernardini, MQ 269

Berney, DM 1137

Beroukhim, R 1218

Berrington de Gonzalez, A 1630

Berteloot, P 244

Berthold, F 1481

Bertrand, F 473

Beskow, C 816

Beusterien, KM 387

Bhuvaneswari, R 1580

Bibeau, F 951

Bicknell, R 957

Bièche, I 473, 1456

Bigner, DD 1986

Bihl, M 1382

Bilim, V 2005

Billadeau, D 2005

Birch, JM 1939

Bird-Lieberman, EL 1

Birgersdotter, A 1393

Bischiniotis, T 734

Bitterman, PB 424

Björk, A 1233

Björkholm, M 1393

Black, KL 303

Black, MA 822

Blanco, I 1456, 1469

Blank, SV 2048

Blay, J-Y 7

Blockley, LY 1724

Blomquist, E 1995

Blomqvist, P 1919

Blows, FM 1338

Bluff, JE 666

Blumm, N 749

Board, RE 1724

Bodmer, WF 1758

Boeck, S 1853

Boender, P 312

Boettcher, HD 1853

Boezen, M 149

Boggess, JF 2048

Bognar, L 722

Böhling, T 1444

Boidot, R 1357

Boissière-Michot, F 951

Bokaee, S 549

Boldrini, L 1869

Bolea-Murga, V 860

Boneberg, E-M 605

Bonifaci, N 1469

Bonnier, P 48

Boot, H 1505

Bordignon, C 219

Bordoni, A 1925

Borena, W 1202

Borovoi, I 1194

Bosch, X 865

Bosserhoff, AK 551

Bottai, M 1932

Boualga, K 1207

Bouaouina, N 1207

Bouffet, E 722

Bougnoux, P 232, 1978

Bouvier, A-M 215

Boven, E 1224

Bowden, DJ 1522

Box, HC 452

Boxus, M 1497

Boyer, JC 441

Boysen, T 530

Brabin, L 1502

Brahimi, S 7

Brahmer, J 1543

Brandt, L 1919

Brash, D 379, 1490

Bratt, O 1233

Braun, Y 80

Brawner, CM 1740

Brenner, H 160

Brewer, C 1456

Brewster, D 541

Brewster, W 1805

Briaire-de Bruijn, IH 1909, 2068

Bridgewater, J 621

Briffod, M 473

Brinkman, A 1824

Brinton, LA 178

Brismar-Wendel, S 511

Brittain-Dissont, S 1290

Britton, PD 1522

Brodbelt, A 124

Broeckaert, MAM 1011

Broglio, K 1813

Brookes, KE 55

Brown, CH 1522

Brown, LG 263

Brown, NJ 666

Brown, R 410

Brugge, J 1699

Brunet, J 2048

Brünker, P 1758

Bruns, CJ 1853

Budzinski, EE 452

Buechner-Steudel, P 1846

Buermeyer, AB 269

Buffart, TE 1011

Bui, B 7

Bulten, J 27

Buonadonna, A 734

Burger, HU 1961

Burgess, R 1543

Bussink, J 372

Butler, J 829

Buttyan, R 951

Buys, S 2048

Buza, N 335

Byrne, AT 1565

Byrne, SM 483

Caballero, G 80

Cairns, DA 1175

Cairns, J 379, 1490

Caldas, C 875, 1338

Caldés, T 1469, 2048

Caligaris Cappio, F 219

Caligo, MA 2048

Callanan, JJ 1565

Calleary, J 19

Calvo, A 1876

Campbell, F 1145

Campbell, HE 1074

Campbell, MJ 1860

Campion, L 166

Campone, M 166, 1995

Campos, M 80

Canestrari, E 715

Canner, JA 774

Cantarini, MV 1724

Capoen, A 244

Cargnelutti, M 335

Carlsen, E 1282

Carney, ME 1805

Carpentier, S 48

Cartledge, J 1175

Carvalho, B 707

Casagrande, F 335

Cascinu, S 1261

Casella, C 2048

Cassidy, J 410, 1033

Cassini, C 2048

Castleberry, AW 882

Castro, F 1329

Catalano, V 715

Cats, A 1505

Causeret, S 1357

Cavaco, BM 1782

Cazorla, A 1469

Celli, JP 2015

Chabaud, S 673

Chalub, FACC 1130, 2066

Chan, A 232

Chan, AT 916

Chan, DW 1433

Chan, FKL 691

Chan, S 403, 1321

Chao, K-Y 174

Chao, R 1543

Charalambous, MP 106

Charbonnel, C 166

Charfi, S 48

Charpin, C 48

Chatterjee, A 822

Chau, H 1555

Chau, I 1033

Chauvenet, M 215

Che, C-M 342

Chen, C 1805

Chen, C-A 174

Chen, C-J 174

Chen, C-Y 738

Chen, F 327

Chen, H 432, 1114

Chen, LL 749

Chen, X 1456, 1805, 2048

Chen, Y-Y 174

Chenevix-Trench, G 1456, 1805, 2048

Cheng, VYY 691

Cheung, ANY 1433

Cheung, PKF 691

Chiappori, A 1044

Chiba, N 1972

Chin, WWL 1580

Chiu, PM 1433

Chodkiewicz, C 1162

Choi, E 504

Chou, C-H 1555

Chouchane, L 1207

Chow, W-H 855

Christakis, NA 749

Christensen, IJ 992

Chua, W 998

Churchill, AJ 1183

Citterio, G 219

Claes, K 2048

Clark, J 1137

Clark, TS 2055

Clarke, SJ 998

Clement, P 1995

Cleton-Jansen, A-M 1909, 2068

Clifford, GM 202, 865

Cocco, E 335

Cohen, S 2048

Coindre, J-M 7

Colas, C 1456

Cole, M 256

Cole, T 1456

Coleman, RE 561, 568

Collet, N 1417

Concin, H 1202

Conrad, CA 615

Conte, P-F 232

Conti, S 1085

Cook, G 1225

Cook, J 1456, 2048

Cook, M 1456, 2048

Cook, MB 855

Cooney, KA 2043

Cooper, CS 1137

Coppolani, E 951

Corbex, M 1207

Cordelières, FP 473

Corey, E 263

Corey, SJ 38

Corrie, P 621

Corsi, V 1869

Cos, S 1613

Costello, E 1145

Couch, FJ 1461, 2048

Coudert, B 1357

Couet, C 1978

Coulet, F 1456

Coulson, S 1692

Coulthard, S 256

Coupier, I 1456

Cowan, RA 582

Cowen, RL 1290

Cox, DG 673

Cramer, DW 1805

Craven, RA 1175

Crawford, DH 1019

Crawford, SM 897

Cremolini, C 374, 715

Crialesi, R 1085

Crijns, APG 149

Cristofanilli, M 1813

Croce, CM 743

Crooks, D 124

Cross, AJ 178

Cross, K 1860

Cross, SS 666, 1692

Cross, W 1839

Crouthamel, M-C 1717

Cruger, DG 2048

Cucci, E 1066

Culine, S 951

Cunningham, D 1033

Cunningham, JM 1461, 1805

Curran, KM 1565

Currid, CA 278

Curtin, NJ 256

Cuzick, J 1137

Dagan, E 2048

Dahiya, R 1374

Dahl, O 1282

Dahmoul, S 1207

Daladimos, T 1059

Dalgleish, AG 803

Daly, M 2048

Damber, J-E 1233

Dame, C 1481

Damery, S 250

Danese, E 548

Dang, NH 983

Dangles-Marie, V 473

Darby, S 1891

Dargere, D 473

Daskalaki, A 589

Daud, A 1044

David, M 215

Davidson, R 1456

Davies, EA 198

Davies, J 549

Davis, M 390

Dawidzik, JB 452

Dawnay, A 160

Dawood, S 1813

Dawson, SJ 1338

De, A 424

de Alava, E 80

de Almeida, VH 782

de Bie, RP 27

de Bock, GH 149

De Cecco, L 1469

De Diego-Flores Chapa, J 860

De Giorgi, U 1813

de Graeff, P 149

de Hullu, JA 27

de Jong, DJ 1671

de Jong, S 149

de Klein, A 813

de Koning, HJ 1833

de la Hoya, M 2048

de la Taille, A 951

de Sanjose, S 865

de Vries, EGE 149

DeAngelis, S 774

Dearden, SP 1724

De-Bono, J 1137

Deen, S 1321

Deißler, H 2048

Deisboeck, TS 749

Deissler, H 1456

Dekker, E 1274

Del Campo-Martinez, MA 860

del Puerto, O 1033

Dell’Omodarme, M 1869

Delprado, W 1345

Demange, L 1456

Dembélé, D 1357

den Dekker, AT 1824

Denis, M 1417

Denny, A 1329

der Kinderen, A 1833

Dering, J 1699

Desjardins, A 1986

Desjardins, L 312

deSouza, NM 1225

Devesa, SS 855

Devi, GR 269

Dhillon, H 998

Di Fabio, F 1261

Díaz-Laviada, I 940

DiCioccio, RA 1461, 1805

Diem, G 1202

Dietrich, K 1316

Díez-Torre, A 64

Dijkstra, G 1671

Ding, K 774

Ding, X 269

Ding, YC 2048

Dingli, D 1130, 2066

Diosdado, B 707

Dirix, LY 1028

Dirks Mulder, A 312

Dishon, S 1456

Ditsch, N 2048

Dive, C 55, 1290, 1724

Dixon, JM 1253

Dixon, L 621

Dodson, AR 1137

Doherty, GA 483

Doherty, JA 1805

Doi, R 908

Doki, Y 1298, 1664

Domchek, SM 1456, 2048

Donovan, JL 390

Doré, JJE 1107

Dorssers, LCJ 1824

Douglas, F 2048

Douglas, JA 2043

Douno, K 202

Downes, CS 441

Draisma, G 1833

Dresemann, G 1995

Driver, KE 1338

Drost, RM 1651

Drucker, L 1402

Dubourg, C 1417

Dudderidge, T 19

Dudek, AZ 1114

Duffaud, F 7

Duffy, SW 881, 1338

Dunn, J 124

Dunne, P 441

Dupont-Lampert, V 605

Durán, M 1456

Durrant, LG 1321

Dutt, A 1218

Easaw, J 1995

Easton, DF 1456, 2048

Eaton, D 1860

Ebert, MPA 691, 699

Eden, TOB 1939

Edlund, CK 1805

Edmondson, RJ 498

Edvardsen, T 575

Edwards, R 1805

Eeles, R 1456, 2048

Egawa, S 908

Egeler, RM 1909, 2068

Eggener, S 2057

Egorin, M 1044

Eide, TJ 1282

Eisenacher, M 80

Eiser, C 561, 568

el Filali, M 312

Elattar, A 498

Elkahwaji, JE 1740

Elliott, J 541

Ellis, CE 1676

Ellisen, LW 1606

Ellison, G 1724

Ellstrøm-Engh, M 534

El-Sahwi, K 335

Elton, P 1502

Emberton, M 19, 2056, 2059

Emile, J-F 7

Enderling, H 1030

Endo, M 225

Engebraaten, O 1307

Engel, C 1456, 2048

Engers, R 1410

Epstein, E 537

Ernberg, I 1393

Eskild, A 534

Espie, M 232

Estlin, EJ 55

Ettinger, SL 1731

Eustace, AJ 1290

Evans, DG 1456, 2048

Evans, JMM 1199

Evans, L 621

Evans, TRJ 410

Fagotti, A 1066

Fahlke, J 1846

Fairley, L 1839

Faivre, J 215

Fajardo-Gutierrez, A 860

Falcone, A 374, 715, 1261

Falk, S 621

Fan, X 303

Farber, RA 441

Farkas, DL 303

Farmer, J-P 722

Fauquette, V 637

Faur, N 350

Faury, D 722

Feinmesser, R 1194

Feng, B-J 1207

Fenwick, K 973

Fergelot, P 1417

Ferguson, RE 1175

Fernandez, PL 1248

Fernández-Ramires, R 1469

Ferrandina, G 1066

Ferrasson, MN 1978

Ferreira, CG 782

Ferrucci, LM 178

Fiebig, B 2048

Field, JK 139

Fietkau, R 1853

Finkelstein, D 465

Finn, RS 1699

Fisch, P 1513

Fischer, JW 2038

Fischer, M 1481

Fisher, AD 498

Fisher, G 1137

Fitzgerald, DJ 483

Fitzgerald, RC 1

Fitzpatrick, P 1900

Fitzpatrick, PA 278

Fleig, WE 1846

Flentje, M 1853

Flockhart, RJ 1448

Flores-Lujano, J 860

Floriani, I 374, 715

Fluge, Ø 1282

Fockens, P 1274

Fodstad, Ø 1307

Foekens, JA 1824

Font, A 1248

Fontaine, J-F 132

Fontanini, G 715, 1869

Forbes, NS 1683

Foretova, L 2048

Forman, D 897, 1269, 1839

Fornander, T 902

Fortuny, J 1316

Fosså, A 575

Fosså, SD 575

Foster, CS 1137

Fostira, F 32

Fountzilas, G 32

Fox, SB 1168, 1749, 1806

Fraise, J 1357

Franc, B 132

Franceschi, S 202, 865

Franchi, M 548

Franke, AA 185

Frattini, M 376

Freed, E 465

Freeman, A 19

Freund, HG 452

Friborg, J 530

Fricker, J-P 2048

Fridley, BL 1461

Friedman, AH 1986

Friedman, E 2048

Friedman, HS 1986

Froberg, M 511

Frost, D 2048

Frost, JA 38

Froster, UG 1456, 2048

Frova, L 1085

Fu, SB 327

Fuchs, CS 465, 916

Fuentes-Pananá, EM 860

Fujimoto, J 377

Fujisawa, M 1731

Fujita, Y 1537

Fujiwara, Y 1298, 1529, 1676

Fujiyoshi, N 967

Fukuda, T 12

Fukushima, M 1537

Fumoleau, P 1357

Furlong, F 1900

Fürstenberger, G 605

Furukawa, J 1731

Furuse, K 1537

Füzesi, L 605

Gaballah, K 418

Gagnon, RC 1676

Gaillard-Kelly, M 71

Galitovskiy, V 1805

Gallagher, R 1620

Gallagher, WM 1565

Gallardo, E 1248

Galluccio, N 715

Galustian, C 803

Gamoh, M 1972

Ganai, S 1683

Gao, G 1699

Gao, H 2030

Garami, M 722

Garbe, C 813

Garcia, MJ 1469

Garcia, S 48

Garcia-Alfonso, P 1039

Gardener, T 582

Garganese, G 1066

Gariboldi, M 1469

Garnier, J 1357

Garzon, R 743

Gascon, P 1248

Gatter, KC 1749

Gavin, A 541

Gavish, M 1194

Gayet, B 473

Gayther, SA 1461, 1805

Geisler, J 1253

Gelderblom, H 357, 1909, 2068

Gentry-Maharaj, A 1461, 1805

Georgoulias, V 465, 589

Geppert, B 537

Geraci, M 1939

Gerdes, A-M 2048

Gershoni-Baruch, R 2048

Geršl, V 792

Giannetta, L 1261

Giaquinta, S 1261

Gijtenbeek, A 1995

Gil, M 1995

Gil-Bazo, I 1876

Gilbert, MR 615

Gill, AJ 1011

Gille, HJP 1456

Ginther, C 1699

Giovannucci, EL 916, 1932

Giraud, S 1456, 2048

Giraudeau, B 1978

Gitsch, G 1513

Giurescu, M 1241

Giusiano, S 48

Glas, R 1699

Gleave, ME 1731

Glenn, WK 1345, 1351

Glynne-Jones, R 742

Gnanapragasam, VJ 1891

Go, MYY 691

Godwin, AK 2048

Gogas, H 32

Goldgar, DE 1207, 2048

Goldschmidt, H 1051

Goldstein, D 998

Gollins, S 621

Gollins, SW 924

Gómez García, EB 2048

Gomi, K 225

Gontarek, RR 1717

González, A 1613, 1876

Gonzalez-Flores, E 1039

Gonzalgo, M 2057

Goode, EL 1461, 1805

Goode, V 582

Gooderham, NJ 106

Goodman, MT 185, 1805

Gordower, L 1995

Gorman, S 1900

Gorodezky, C 860

Gottgens, B 741

Gouraud, W 166

Govindan, R 1543

Grabsch, HI 1011

Gralec, R 734

Grande-Pulido, E 1876

Grandoch, M 2038

Grannis Jr, FW 882

Graubard, BI 178

Gravdal, K 1282

Gravekamp, C 1329

Gray, AM 1074

Graziano, F 715

Green, C 238, 2062

Green, S 1995

Greenberg, DC 1338, 1522

Greene, MH 1456, 2048

Greenfield, DM 561, 568

Gregorc, V 219

Grelier, G 673

Greulich, H 1218

Griggs, J 1807

Groves, MD 615

Gruis, NA 312

Gschwantler-Kaulich, D 2048

Gu, YS 1635

Guchelaar, H-J 357

Guenther, T 742

Guidi, GC 548

Guillén-Grima, F 1876

Gunčová, I 792

Gurpide, A 1876

Gururangan, S 1986

Gwack, J 526

Haanen, JBAG 1224

Haba, M 202

Hader, C 1410

Hadfield, JA 829

Häggman, M 1233

Hahnfeldt, P 1030

Haines, R 1692

Hajjaji, N 1978

Halbert, G 1860

Hall, GL 139

Hall, M 1565

Halvorsen, TB 1282

Hamamoto, R 98

Hamann, U 1456, 2048

Hamdi-Cherif, M 1207

Hamdy, F 390

Hamilton-Dutoit, S 530

Hampson, IN 829

Hampson, L 829

Hamzany, Y 1194

Han, C 1749

Han, DJ 1658

Hancock, BW 561, 568

Handy, B 1813

Hannaoui, N 1248

Hansen, AV 530

Hansen, TvO 2048

Hanzely, Z 722

Hao, J 432

Haque, T 1019

Harada, H 225

Harden, S 410

Harmey, JH 278

Harper, SJ 1183

Harrington, KJ 549

Harris, AL 742, 1074, 1749

Harris, MA 582

Harrison, M 1900

Hartig, SM 38

Hasan, T 2015

Hasenbrink, G 160

Hasenburg, A 1513

Hašková, P 792

Hatina, J 1410

Hatori, T 908

Hattori, Y 1884

Hatzidaki, D 589

Hau, P 1995

Haugan Moi, LL 1253

Hauglid Flågeng, M 1253

Hauke, RJ 1740

Hauser, P 722

Havelange, V 743

Havre, PA 983

Hawkins, C 722

Hayes, RB 522

Haylock, B 124, 924

Haynes, R 897

Hazama, S 1374

He, XT 829

Healey, S 1456, 2048

Heaney, J 1316

Heijnsdijk, EAM 1833

Heikkinen, T 2048

Heinemann, V 1853

Hemminki, K 935, 1091, 1792

Henderson, BE 185

Heng, B 1345, 1351

Henry, JY 803

Herbert, JMJ 957

Herbst, M 1853

Herndon, JE 1986

Herrero, R 865

Herrero-Martín, D 80

Hersey, P 387

Hess, KR 615

Higaki, K 598, 1031

Higham, SE 666

Highley, M 621

Hill, A 1620

Hills, A 418

Hinke, A 1853

Hinkula, M 1213

Hinoda, Y 1374

Hinz, U 457

Hirahara, F 2023

Hiraike, H 145, 1220

Hiraike-Wada, O 145, 1220

Hirakawa, A 1529

Hirakawa, K 1100, 1365

Hirata, H 1374

Hirata, T 1529

Hiripi, E 1792

Hirose, H 1664

Hirotsu, M 2030

Hjerpe, A 511

Hlatky, L 1030

Hobbs, R 250

Hodgson, S 2048

Hoefer, MM 605

Hoene, V 1481

Hogdall, CK 1805

Hogdall, E 1461, 1805

Hogendoorn, PCW 1909, 2068

Hogervorst, FBL 1456, 2048

Hogg, A 55

Holgersson, Å 816

Hollenbeck, AR 1630

Hollis, BW 916

Holt, SK 1805

Holt, SV 55

Holte, H 575

Homer, J 139

Hommes, DW 765, 1671

Hong, D 1798

Hong, K-L 1699

Hong, Y-C 526

Hoos, A 387

Hooshmand, S 465

Hopper, JL 2048

Hopwood, P 582

Horai, T 225

Horenblas, S 1505

Horgan, K 106

Hori, D 967

Horiike, A 225

Hose, D 1051

Hosokawa, S 145, 1220

Hostein, I 7

Hotta, K 1709

Hou, H-A 738

Howell, A 582

Howell, SJ 582

Høyer-Hansen, G 992

Hoyle, M 238, 2062

Hozawa, A 849

Hroch, M 792

Hsiao, M-L 174

Hsieh, C-Y 174

Hu, J 303

Hu, Y 1798

Huang, H-L 1555

Huang, W-Y 522

Huang, Z 269

Huet, G 637

Hughes, A 1724

Hughes, DJ 1456

Hughes-Davies, L 875

Humar, B 822

Hunter, K 615

Hurme, S 1005

Husband, D 124

Hussein, D 55

Hutzen, B 774

Hwang, JY 303, 1658

Iacopetta, B 998

Ides, J 628

Iezzi, G 1382

Iijima, H 452

Ijichi, M 967

Illing, R 19

Imai, K 1374

Imyanitov, EN 2048

Inaji, H 598, 1031

Ingvar, C 1817

Inoue, Y 1648

Ip, NY 691

Ishii, H 1664

Ishioka, C 1972

Islami, F 1641

Ismail, M 549

Ismail, T 250

Ito, T 598, 1031

Ito, Y 1676

Ivanova, A 1481

Iversen, ES 1805

Iveson, T 621

Iwata, H 1676

Izumi, M 287

Jaakkola, PM 373

Jabado, N 722

Jacob, K 722

Jacobs, IJ 160

Jacobson, BA 424

Jacques, C 132

Jager, MJ 312

Jalbout, M 1207

Jansen, JBMJ 1274

Jay-Dixon, J 424

Jendrossek, V 2038

Jenkins, RE 1145

Jennison, E 1805

Jernström, H 1817, 2048

Jézéquel, P 166

Jin, HC 691, 699

Jin, Y 327

Joensuu, H 1444

Johannsson, OT 2048

Johansson, B 511

Johansson, J-E 1932

John, EM 2048

Johnson, GR 2043

Johnson, KJ 518

Johnson, PJ 295, 621

Jonckheere, N 637

Jones, A 840

Jones, AM 1269

Jones, AP 897

Jones, E 621

Jonkers, J 1651

Jönsson, P-E 1817

Jooste, V 215

Jouan, F 1417

Jouve, J-L 215

Joyce, K 124

Jubb, AM 1749

Judson, I 1860

Jütting, U 1513

Kabba, I 202

Kage, M 967

Kahlert, C 457

Kai, M 363

Kaizaki, R 1100

Kakazu, KK 185

Kakizaki, M 849

Kakudo, Y 1972

Kakuma, T 967

Kalinoglou, N 1059

Kamakari, S 32

Kamangar, F 1641

Kamura, T 967

Kanehiro, H 1709

Kanemitsu, K 908

Kang, D 526

Kanieff, M 1085

Kanno, J 350

Kano, M 1425

Kanter, L 816

Karabelis, A 1059

Karagas, MR 1316

Karakitsos, P 1382

Karamitopoulou, E 1382

Karra, H 1005

Karvounis, N 1059

Kasamatsu, A 684

Kato, S 1972, 1972

Katsumata, N 1529

Katsura, K 1676

Kaufman, B 2048

Kauppila, A 1213

Kaur, S 1155

Kawaguchi, S 1425

Kawaguchi, T 1537

Kawahara, M 1537

Kawamura, T 287

Kawana, K 145, 1220

Kawano, K 967, 1884

Kawazoe, H 2005

Kay, EW 483

Kearins, O 395, 1032

Keita, N 202

Kelly, JD 98

Kelsey, KT 1316

Kemp, T 829

Kemsley, KR 1724

Kennedy, J 582

Kenngott, HG 457

Ketzer, P 256

Key, TJ 192

Khan, OA 550

Kheuang, L 951

Khyatti, M 1207

Kiewe, P 1241

Kikuchi, K 2023

Kikuchi, M 116, 492

Kilic, E 813

Kim, C-S 526

Kim, H 1664

Kim, J-E 1658

Kim, MA 504

Kim, M-H 1658

Kim, S 1162

Kim, SC 1658

Kim, TW 1658

Kim, Y 526

Kimura, S 1425

King, AG 1717

Kirchhoff, T 2048

Kirkham, A 19

Kirkland, SC 320

Kishida, Y 1537

Kitchener, HC 829, 1502

Kitteringham, N 1145

Kjaer, SK 1461, 1805

Kjekshus, J 575

Kjellevold, K 1282

Kleber, G 1846

Klein, B 1051

Klenk, J 1202

Klinger, R 1900

Klymenko, T 55

Ko, K-P 526

Koch, M 457

Kodama, Y 1365

Kodera, Y 492

Koelink, PJ 765

Kogera, FA 139

Koh, J 598, 1031

Kohno, N 598, 1031

Koizume, S 2023

Koljonen, V 1444

Kolonel, LN 185

Komichi, H 598, 1031

Komiya, S 2030

Komuta, K 1537

Kondo, T 1425

Konecny, GE 1699

Konerding, MA 64

Konishi, N 1709

Konstantopoulou, I 32, 1456

Kontopodis, E 589

Kontorovich, T 2048

Koong, S-L 174

Koreckij, T 263

Korkeila, E 373

Kosarin, K 2048

Koschel, A 1574

Koscielny, S 902

Kosmas, C 1059

Kosuge, T 908

Kotapati, S 387

Koulibaly, M 202

Kouno, T 1529

Koyama, H 598, 1031

Kozuka, T 225

Kratzke, MG 424

Kratzke, RA 424, 1114

Kronqvist, P 1005

Kubota, K 1537

Kucukmetin, A 498

Kuenen, M 1505

Kukko, H 1444

Kulke, MH 465

Kumar, R 1717

Kunitoh, H 736, 1549

Kuopio, T 1005

Kuramoto, H 145, 1220

Kuribayashi, H 1884

Kuriyama, S 849

Kuss, O 1846

Kusunoki, M 295, 1648

Kwak, E 465

Kweekel, DM 357

Kwok, TT 699

Kynaston, H 390

Kyono, S 598, 1031

Kyyrönen, P 1213

Laborde, AL 1456

Lafaras, C 734

Lagord, C 395, 1032

Laheij, RJF 1274

Lai, M-Z 1555

Lai-Kuen, R 473

Laitman, Y 2048

Lalloo, F 1456, 2048

Lam, A 160

Lambiase, A 219

Lambrechts, HAJ 628

Lambros, MBK 973

Lamers, CBHW 765

Lampkin, S 1329

Landberg, G 1769

Lane, JA 390

Lange, EM 2043

Lardon, F 628

Laroche-Clary, A 350

Lasset, C 673

Lassmann, S 1513

Lavaut, M-N 48

Lawrence, G 395, 1032

Lawson, JS 1345, 1351

Le Cesne, A 7

Le Floch, O 1978

Le Marchand, L 185

Le Morvan, V 350

Leach, MO 1860

Leal, B 1329

Leclair, F 166

Lecomte, R 1565

Lee, CK 998

Lee, CP 1860

Lee, FY 38

Lee, H 504

Lee, J-H 1044

Lee, J-L 1658

Lee, JS 1658

Lee, K-Y 327

Lee, SK 1658

Lee, SS 1658

Lee, WYW 1433

Lee, Y-K 504

Leek, R 1749

Legler, DF 605

Leite, V 1782

Leitzmann, MF 522

Lejbkowicz, F 1456, 2048

Léoné, M 1456

Leong, C-O 1606

Léon-Goddard, S 673

Lepage, C 215

LeQuesne, J 1338

Leroy, IM 1824

Lestuzzi, C 734

Leunen, K 244

Leung, HY 1891

Leung, WK 691

Levin, AM 2043

Levin, RA 1717

Levin, VA 615

Levine, E 924

Levitt, NC 550

Levy, AR 387

Lewensohn, R 816

Li, H 1635

Li, JJ 691, 699

Li, J-L 1749

Li, Q 71

Li, W 342

Li, X 1792

Li, Y 432

Lichtenberg-Frate, H 160

Lidereau, R 1456

Liebow, M 1461

Lien, EA 1253

Lightfoot, T 106

Lilleng, R 1282

Liloglou, T 124, 139

Lim, LY 1606

Lin, J 774

Lin, L-I 738

Linabery, AM 518

Lindell, M 871

Lindemann, K 534

Lindemann, S 1241

Lindor, N 2048

Lindqvist, PG 537

Liniker, E 658

Linton, K 582

Lippi, G 548

Lishner, M 1402

Liu, G 303

Liu, GY 327

Liu, Q 342

Liu, VH 615

Liu, VWS 1433

Liu, WM 803

Liu, XZ 1635

Liu, Z 238, 2062

Lizard-Nacol, S 1357

Llort, G 1469

Lobel, B 1417

Lofts, F 621

Loizidou, M 658

Lomholt, AF 992

Longatto-Filho, A 973

Lønning, PE 1253

Lopes, JM 973

Lorenzo Bermejo, J 1091, 1792

Lorenzo, JRM 1248

Loud, JT 1456

Loughery, J 441

Loupakis, F 374, 715

Loussouarn, D 166

Lovat, PE 1448

Lowe, D 139

Lu, S 1329

Lu, W 1798

Luccarini, C 1456, 2048

Lucchi, M 1869

Lugli, A 1382

Lund, M-B 575

Lundgren, K 1769

Lundin, C 91

Lurie, G 1805

Lutgens, MWMD 1671

Lutze-Mann, L 1345, 1351

Luyten, GPM 312

Lyerly, HK 269

Lynch, HT 1456, 2048

Ma, X 178

Maachi, F 1207

Maat, W 312

Määttänen, L 843

Mackay, A 973

Mackintosh, C 80

MacRobert, AJ 658

Maddams, J 541

Madoz-Gúrpide, J 80

Maeda, T 492

Magnani, M 715

Mai, P 2048

Mai, Z 2015

Maillé, P 951

Maitani, Y 1884

Majois, F 232

Makin, GWJ 55

Makino, T 1298

Malagarie-Cazenave, S 940

Malamos, N 1059

Malmer, B 2048

Maloney, SL 957

Malthiery, Y 132

Mancer, K 1580

Manivong, K 1699

Mann, JI 192

Manoukian, S 1456, 2048

Marangoni, E 473

Maraveyas, A 621

Marcello, JE 1986

Marchion, D 1044

Margariti, A 1066

Margison, GP 550

Mårlind, J 645

Marsit, C 1316

Martin, A 295

Martin, RM 390

Martinez-Avalos, A 860

Martínez-Campa, C 1613

Martinho, O 973

Martins, A 973

Martins, AS 80

Martoni, AA 1261

Marwah, S 465

Masi, G 715

Mason, L 924

Masson, D 1417

Massonnet, G 473

Massuger, LFAG 27

Masuda, R 1365

Mather, S 1183

Mathers, ME 1891

Mathews, M 1107

Matsunoshita, Y 2030

Matsuoka, T 1100

Matsuura, N 1298

Matsuyama, Y 908

Matsuzaki, T 1100

Matterne, U 457

Maurel, J 1162

Mavroudis, D 589

Maw, MK 377

Maxwell, PJ 1620

Mayne, ST 178

Mazurová Y 792

Mazzucchelli, L 376, 1925

McArdle, CS 557

McAulay, KA 1019

McCall, JL 822

McCann, A 1900

McCann, GP 403

McCann, R 1502

McCarty, CA 178

McDaid, JR 441

McDonald, PJ 742

McDonald, SL 209

McEvoy, JW 2064

McGee, S 1565

McGown, A 829

McGuffog, L 1456, 2048

McGuire, V 1805

McKenna, SL 1585

McLendon, RE 1986

McMillan, DC 557, 1650

McNamara, J 1860

McNoe, L 822

McWalter, G 1724

Mediavilla, MD 1613

Medina-Sanson, A 860

Mego, M 1813

Meijer, CJLM 202

Meijer, GA 707, 1011

Meijerink, WJHJ 707

Meijer-van Gelder, ME 1824

Meijnders, P 628

Meindl, A 1456, 2048

Meinhold, CL 1630

Meira, DD 782

Mejía-Aranguré, JM 860

Melbye, M 530

Melchers, WJG 27

Melero, I 1876

Melia, J 1269

Mellado, B 1248

Mellgren, G 1253

Menakuru, SR 666

Mendez-Ureña, M 1039

Meng, XN 327

Menon, U 160, 1461, 1805

Mesli, S 1207

Messiou, C 1225

Metcalfe, C 390

Meyer, B 803

Meyer, T 621

Meyerhardt, JA 465, 916

Meyers, C 615

Meyerson, M 1218

Mezhybovska, M 1596

Michael, M 550, 998

Michel, G 561, 568

Mickisch, G 2060

Middleton, MR 387, 550

Midgley, R 550

Miethe, S 1853

Mikaelian, I 673

Mikami, T 116, 492

Miki, C 295, 1648

Milanezi, F 973

Miller, WR 1253

Mills, J 403

Milne, RL 2048

Mimori, K 1664

Minato, K 1537

Minelli, G 1085

Minn, H 373

Minowa, T 1884

Minton, S 1044

Mio, T 1537

Mirebeau-Prunier, D 132

Miron, A 2048

Mirtsos, C 1555

Mitchell, P 998

Mitomori, S 1374

Mitsuyama, S 598, 1031

Miwa, A 1100

Miyagi, E 2023

Miyagi, Y 2023

Miyake, H 1731

Miyamoto, Y 145, 1220

Miyata, H 1298

Miyoshi, N 1664

Mizuuchi, E 1365

Moehler, M 1846

Moeller, HC 1749

Moerman, Ph 244

Mogler, C 457

Mohammadi, M 530

Mohd, AB 269

Mohri, T 295

Mohri, Y 295, 1648

Molinari, F 376

Mollberg, N 457

Møller, H 198, 541, 1137

Momi, N 1155

Moncoutier, V 2048

Monden, M 908

Mongera, S 707

Mongrain, K 1565

Monisvais, D 38

Montagna, M 2048

Montagnana, M 548

Montes, JL 722

Montpetit, A 722

Monypenny, I 395, 1032

Moore, C 658

Moore, SC 522

Moorman, PG 1805

Moran, A 1939

Morand, M 232

Mordenti, G 1051

Moreaux, J 1051

Morgan, R 549

Morgentaler, A 1949

Mori, M 1298, 1664

Morimoto, C 983

Mororó, JS 782

Morota, M 225

Morris, AD 1199

Morris, C 1724

Morris, E 1269

Morris, J 924

Morrison, PJ 2048

Mortensen, NJ 1749, 1758

Mortimer, P 550

Mosetto, M 1900

Moss, SM 1269

Mössner, E 1758

Motoyama, T 2005

Moxham, M 124

Moxham, T 238, 2062

Moyes, L 1650

Moyret-Lalle, C 673

Moysich, KB 1805

Mueller, DW 551

Mukai, K 287

Mukherjee, A 1321

Mukherjee, J 387

Muller, D 2048

Müller, M 1410

Muñoz, N 865

Muñoz-Martin, A 1039

Munster, PN 1044

Murakami, H 225

Murase, M 1425

Murchison, JT 840

Murphy, SK 269

Murphy, SP 185

Murphy, T 1891

Murray, FE 483

Murray, PG 1393

Murtagh, J 1565

Murtola, TJ 843

Mussi, A 1869

Muto, A 2005

Mutri, V 1261

Myint, S 924

Mylonakis, N 1059

Nachtigal, P 792

Nagae, G 145, 1220

Nagai, M 849

Nagao, H 2030

Nagel, G 1202

Nagler, RM 1194

Nagtegaal, ID 372

Nagy, Z 957

Naito, S 2005

Nakagawa, K 1549

Nakagawa, S 145, 1220

Nakajima, K 1298

Nakajima, Y 1709

Nakamura, K 1100

Nakamura, S 1676

Nakamura, Y 98, 225, 2023

Nakano, K 1537

Nakao, A 908

Napoletano, S 278

Naredi, P 91

Nasri, S 822

Nathanson, KL 1456, 2048

Navarro, P 1145

Neal, DE 98, 390, 1491

Near, AM 1805

Nedjadi, T 1145

Nei, T 145, 1220

Neilan, TG 483

Neoptolemos, JP 1145

Neri, D 645

Ness, RB 1805

Nestorov, I 1051

Neuhausen, SL 2048

Nevanlinna, H 1456, 2048

Neven, P 244

Ng, EKO 691, 699

Ng, K 916

Ng, SSM 699

Ng, YP 691

Ngan, HYS 1433

Nguyen, H 263

Nicolaiew, N 951

Niederacher, D 2048

Nielsen, HJ 992

Nieminen, A 1005

Nikolova, Z 1995

Nilsson, B 816

Nishida, T 1298

Nishikawa, H 598, 1031

Nishimura, T 225, 287

Nishino, Y 849

Nishio, M 225

Nishio, S 967

Nishioka, M 1374

Nishiwaki, Y 1549

Nóbrega, I 782

Noda, K 1549

Noguera-Troise, I 1749

Nogues, C 1456

Nomi, T 1709

Nordenskjöld, B 1769

Nordle, Ö 1233

Norfleet, J 1986

Norizoe, C 1957

Norman, A 582

Northover, JMA 742

Nortier, JWR 357

Novello, S 1543

Ntouroupi, T 1758

Nuijten, M 2060

Nykänen, M 1005

Oakley, R 418

O’Brien, V 410

Ochiai, K 1957

O’Connor, AE 1565

O’Connor, D 1900

Oda, K 145, 1220

O’Donovan, TR 1585

Offit, K 2048

Ogden, C 19

Ogino, S 465

Ogita, M 598, 1031

Ogston, SA 1199

Ogunleye, AA 1199

Oh, S 1114

Ohira, M 1100

Oh-ishi, M 492

Öhlschlegel, C 605

Öhlund, D 91

Ohmori-Matsuda, K 849

Ohno, Y 287

Ohnuma, K 983, 1365

Ohyanagi, F 225

Oka, M 1374

Okamoto, A 1957

Okamura, K 598, 1031

Okayasu, I 116, 492

Okello, C 198

Okines, A 1033

Oldenburg, B 1671

Olea-Herrero, N 940

Oliver, AW 829

Oliver, CT 2048

Olivier-Faivre, L 2048

Olivo, M 1580

Olsson, H 537

Olver, I 550

Öman, M 91

Omar, MM 139

O’Neill, K 1565

Oosting, J 1909, 2068

Oram, SH 741

Ordóñez, JL 80

Oregioni, A 1860

Orlowska-Volk, M 1513

Orr, MCM 1724

Orsini, N 1932

Osaki, A 598, 1031

Osaki, Y 1549

Osborne, R 621

O’Shea, DF 1565

Osman, A 403

Osorio, A 1456, 1469, 2048

Ostermaier, S 1853

Osterwalder, B 1961

O’Sullivan, GC 1585

O’Sullivan, J 1900

O’Sullivan, NC 278

O’Sullivan-Coyne, G 1585

Osuna, D 80

Otero-Motta, AP 80

Otsuka, K 1972

Otte, AP 1282

Ottley, CJ 1290

Otto, F 605

Ougolkov, A 2005

Oya, M 2005

Ozcelik, H 2048

Pacheco, JM 1130, 2066

Pagano, M 1932

Paglia, A 1066

Pallares, C 1543

Palomo-Colli, MA 860

Pan, J 1635

Panayiotides, I 1382

Pandha, H 549

Panizo, A 1876

Paraskeva, C 1124

Pardal, F 973

Paredes-Aguilera, R 860

Park, BK 1145

Park, DH 1658

Park, N-H 504

Park, P-G 504

Park, SK 526

Park, S-Y 185

Park, Y 598, 1031, 1630

Partridge, M 418

Patard, J-J 1417

Patel, MR 424

Paterson, J 1456

Patrzyc, HB 452

Pattyn, GGO 628

Paul, J 410

Pauwels, B 628

Payne, GS 1860

Pearce, CL 1805

Pearson, DG 1290

Peat, I 403

Pecorelli, S 335

Pedley, RB 645

Peock, S 1456, 2048

Perez-Gracia, JL 1876

Perez-Manga, G 1039

Perez-Saldivar, ML 860

Perez-Vera, P 860

Perkins, J 759

Peros, G 1382

Perraki, M 589

Pertesi, M 32

Peterlongo, P 2048

Pettigrew, J 1620

Peyrat, J-P 2048

Pezzella, F 1749

Pfeiler, G 2048

Pharoah, PDP 1338, 1461, 1805

Phelan, C 1461

Philips, J 465

Phillips, JB 658

Picchi, A 1869

Pichert, G 1456, 2048

Pichot, CS 38

Piedmonte, M 2048

Pierotti, MA 1469

Pignatelli, M 1124

Pigny, P 637

Pinheiro, C 973

Pinto, C 1261

Pita, G 2048

Pita, JM 1782

Piwocka, K 1585

Ploussard, G 951

Plummer, ER 256

Plummer, M 865

Pocard, M 473

Podack, ER 381

Polunovsky, VA 424

Ponder, BAJ 98

Popadiuk, C 1107

Popelová, O 792

Porchet, N 637

Poremba, C 80

Porwit, A 1393

Post, WJ 149

Postma, C 707

Poupon, M-F 473

Powell, P 390

Poynter, JN 518

Prati, MC 1869

Prencipe, M 1900

Prior, C 1876

Pripp, AH 575

Prizada, A 1107

Probst-Hensch, NM 1925

Provenzano, E 1338

Puduvalli, VK 615

Puisieux, A 673

Pujana, MA 1469

Pukkala, E 1213, 1444

Punt, CJA 219, 357

Pyrhönen, S 373

Qi, J 1699

Qi, Y-J 295

Qualtrough, D 1124

Quiben-Pereira, R 1039

Quirke, P 1011

Qureshi, U 645

Rabai, EM 957

Radford, JA 582

Radice, P 1456, 2048

Radnay, J 1402

Rahbari, NN 457

Rai, Y 1676

Rainbird, K 759

Raizer, J 1995

Rallis, G 1382

Ramón y Cajal, T 2048

Ramus, SJ 1461, 1805

Ranson, M 550, 1724

Rapp, K 1202

Rasheed, S 742

Ratner, E 335

Rau, HG 1853

Raum, E 160

Ray, AM 2043

Raynaud, FI 1860

Rayoo, M 1168, 1806

Reardon, DA 1986, 1995

Reddick, R 1329

Redman, V 250

Reed, MW 666

Reeve, AE 822

Refsum, S 1282

Reich, JM 879

Reif, S 1241

Reis, RM 973

Reis-Filho, JS 973

Ren, J-S 1641

Ren, Y 342

Rennel, ES 1183

Rennert, G 1456, 2048

Renshaw, C 198

Requirand, G 1051

Reuben, JM 1813

Reuter, VE 1137

Révillion, F 2048

Reynolds, NJ 1448

Rezaei Kalantari, H 1222

Ricart, AD 1162

Richon, S 473

Ricolleau, G 166

Rider, DN 1461

Riener, M-O 1513

Rimokh, R 673

Rioux-Leclercq, N 1417

Risk, JM 139

Rivera-Luna, R 860

Rizvi, I 2015

Roalfe, A 250

Robert, J 350

Roberts, DL 1290

Roberts, SA 1502

Robinson, D 198

Robison, LL 518

Robson, CN 498, 1891

Röcken, C 699

Rodabaugh, KJ 452

Rodriguez, GC 2048

Rodriguez-Zepeda, MC 860

Rogers, G 238, 2062

Rojas Llimpe, FL 1261

Rokadiya, S 139

Rolfe, I 759

Romeo, S 1909, 2068

Romero-Guzman, L 860

Rominger, DH 1717

Roninson, IB 1900

Roobol, MJ 1833

Rosbrook, B 1162

Rose, A 2038

Rose, C 1817

Rose, M 1051

Rosell, R 1543

Ross, JA 518

Rossi, J-F 1051

Rossing, MA 1805

Rotter, AJ 882

Rouillé, V 1051

Rouleau, E 1456

Rousseau, JA 1565

Rübben, H 2038

Ruddle, R 1860

Ruf, W 2023

Rulli, E 715

Rutherford, TJ 335

Ruzzo, A 715

Ryan, A 418

Ryan, AJ 278

Sader, E 722

Sadiq, AA 424

Sadlier, DM 483

Saigenji, K 116

Saijo, N 736, 1549

Saint-Laurent, N 637

Saito, K 684

Saito, T 598, 1031

Sakayori, N 1972

Sakuma, K 684

Sakuma, Y 2023

Salamanca-Gómez, F 860

Saletti, P 376

Sallustio, G 1066

Saltz, L 1033

Salvatore, L 715

Salvesen, HB 1218

Sampson, JH 1986

Samuel, L 924

Sánchez-Barceló, EJ 1613

Sankila, R 1444

Sanner, K 871

Sanson-Fisher, R 759

Santin, AD 335

Santini, D 374, 715

Santoro, A 219

Santos, FC 1130, 2066

Saridaki, Z 465

Sasaki, H 2030

Sasaki, Y 1676

Sathornsumetee, S 1986

Sato, N 1425

Sato, T 116

Sato, Y 492

Sauer, R 1853

Sauerzapf, V 897

Savagner, F 132

Savulescu, D 1194

Scagliotti, GV 1543

Scambia, G 1066

Scardino, P 1137

Schaefer, D 1456, 2048

Schaefer, K-L 80

Schatzkin, A 1630

Scherhag, A 1961

Schildkraut, JM 1461, 1805

Schinzari, G 1261

Schirmacher, P 457

Schlegel, U 1995

Schliemann, C 645

Schmalenberg, H 1846

Schmidt, C 1758

Schmidt, M 2038

Schmidt, MK 1505

Schmoll, H-J 1241

Schmutzler, RK 1456, 2048

Schned, A 1316

Scholten, T 1853

Schreiber, TH 381

Schultz, H 1995

Schulz, WA 1410

Schwartz, PE 335, 2048

Schwarzer, G 1947

Schwarzer, S 160

Scott, LC 410

Scott, R 19

Scriven, P 1692

Scully, GM 483

Searle, C 582

Seaton, A 1620

Secq, V 48

Seddon, B 1860

Segura, V 1876

Sekimoto, M 1664

Selaru, P 1543

Selby, PJ 1175

Sellers, TA 1461, 1805

Sellers, WR 1218

Semba, S 1365

Senn, H-J 605

Seo, DW 1658

Serre, D 722

Setoguchi, T 2030

Sevillano, V 80

Shah, NC 1491

Shapiro, H 1402

Sharma, NL 1491

Shaw, RJ 139

Sheach, LA 498

Shehata, M 1321

Shen, Y 1513

Shenoy, A 124

Shibata, H 1972

Shibata, K 1537

Shibata, T 1549

Shih, L-Y 174

Shiiba, M 684

Shimada, K 1709

Shimada, M 908

Shimizu, C 1529

Shimodaira, H 1972

Shimojima, A 1957

Shin, A 526

Shino, Y 684

Shinozuka, K 684

Shinto, O 1100

Shiraishi, K 1884

Shirakawa, M 1298

Shirasawa, H 684

Shiroiwa, T 12

Shivdasani, RA 465

Sho, M 1709

Shoji, K 145, 1220

Shpitzer, T 1194

Shu, XO 518

Sibson, R 124

Siebers, AG 27

Siegel, ER 335

Siena, S 1261

Sier, CFM 765

Sieron, P 1410

Siersema, PD 1671

Sieuwerts, AM 1824

Sihto, H 1444

Silasi, D-A 335

Silió, M 64

Silva, A 973

Silva, RLA 782

Silván, U 64

Silver, A 209, 742

Silver, J 465

Silver, M 465

Sim, S 1175

Simard, J 1456, 2048

Simon, G 1044

Simpson, AJ 840

Šimůnek, T 792

Singer, CF 2048

Singh, B 1749

Singh, K 1124

Sinha, R 178

Sinilnikova, OM 1456, 2048

Sinnett, D 1456

Sjöberg, J 1393

Sjölander, A 1596

Skikuniene, J 816

Slamon, DJ 1699

Sleijfer, S 1824

Smedby, KE 1919

Smid, M 1824

Smith, D 882

Snijders, PJF 202

Snowden, JA 561, 568

Sobo, M 774

Socinski, MA 1543

Solé, X 1469

Sollier, C 722

Sone, K 145, 1220

Sone, T 849

Song, H 1461, 1805

Soo, K-C 1580

Soubrier, F 1456

Souglakos, J 465

Sousa, A 1248

Spagnoli, G 1382

Span, PN 372

Spano, J-P 1162

Speirs, V 106

Spencer, EA 192

Spiess, A-C 2048

Spitale, A 1925

Spring, BQ 2015

Springett, GM 1044, 1162

Spurdle, AB 1456, 1805, 2048

Stampfer, MJ 916

Stanley, A 1175

Stasi, I 715

Stathopoulos, E 589

Staton, CA 666

Stechly, L 637

Stecker, K 1574

Steers, G 1749

Stegmaier, C 160

Stein, K 238, 2062

Steininger, H 605

Stella, G 1261

Stenman, UH 843

Štěrba, M 792

Steward, J 541

Stewart, L 1183

Stish, BJ 1114

Stockton, DL 840

Stokkers, PCF 1671

Stolzenberg-Solomon, RZ 1630

Stoppa-Lyonnet, D 1456, 2048

Strand, A 871

Strasak, A 1202

Stratford, IJ 1290

Stretch, R 1502

Studebaker, AW 774

Stumpf, M 1513

Subra, F 7

Suchting, S 957

Suehiro, Y 1374

Sugawara, Y 849

Sugimura, K 2030

Sugino, T 1884

Sullivan, D 1044

Sullivan, DC 957

Sun, RW-Y 342

Sund, M 91

Sundar, S 957

Sundquist, J 935, 1091, 1792

Sundström, J 373

Sung, JJY 691, 699

Susini, C 637

Susnerwala, S 924

Sutter, C 1456

Suzuki, T 598, 1031, 1972

Swindell, R 582, 621, 924

Sylla, BS 202

Symonds, RP 403

Syrjänen, K 373

Szabo, CI 2048

Szabo, SM 387

Tabone-Eglinger, S 7

Tabori, U 722

Tachibana, M 287

Taguchi, T 1676

Taillibert, S 1995

Takahashi, A 1425

Takahashi, D 1957

Takahashi, S 1972

Takahashi, T 225

Takano, EA 1168, 1806

Takashima, S 1676

Takatsuno, Y 1664

Takeda, K 1549

Takemoto, S 967

Takeno, A 1298

Taketani, Y 145, 1220

Takiguchi, S 1298

Talbot, CJ 403

Talbot, IC 742

Talvinen, K 373, 1005

Tam, PK 342

Tamaya, T 377

Tamber, N 973

Tamez, S 1957

Taminiau, AHM 1909, 2068

Tammela, TLJ 843

Tamura, K 1529

Tamura, T 736, 1549

Tan, EY 1749

Tan, S 1860

Tanaka, F 598, 1031

Tanaka, K 1648

Tanaka, M 908

Tanaka, T 1957

Tanteles, GA 403

Tanzawa, H 684

Tartakover-Matalon, S 1402

Taskila, T 250

Tavassoli, FA 335

Taylor, MA 1074

Taylor, PR 1641

Tekkis, PP 742

Tendo, M 1100

Tepikin, A 1145

ter Braak, M 2038

Terada, A 967

Terakawa, T 1731

Teramukai, S 1537

Terhaar Sive Droste, JS 707

Terracciano, L 1382

Terrier, P 7

Terry, KL 1805

Terry, MB 2048

Terry, S 951

Thiel, E 1241

Thodi, G 32

Thomas, H 1891

Thomas, J 1269

Thomas, S 1044

Thomassen, M 2048

Thompson, D 1175

Thompson Coon, JS 238, 2062

Thong, PSP 1580

Thorne, H 1168

Thorogood, M 192

Thurston, G 1749

Tien, H-F 738

Tilby, MJ 1290

Tilling, K 390

Timmer-Bonte, JNH 219

Titus-Ernstoff, L 1805

Tizzoni, L 2048

To, KF 691

To, RMY 1433

Toi, M 1676

Toiyama, Y 1648

Tokuda, Y 1676

Tokuyama, W 116

Toland, AE 2048

Tomita, Y 2005

Tong, J 691

Tonini, G 715

Tonn, J 1995

Topham, C 924

Torigoe, T 1425

Torri, V 374

Toutain, J 350

Tracy, M 1860

Traish, AM 1949

Tran, B 1345, 1351

Travis, RC 192

Treilleux, I 673

Triau, S 132

Tsakonas, G 1059

Tsang, WP 699

Tsavaris, N 1059

Tsuboi, M 1537

Tsuchiya, E 2023

Tsuda, N 967

Tsuji, I 849

Tsukahara, T 1425

Tsutani, K 12

Tsutsumi, Y 1957

Tsuya, A 225

Tubiana-Mathieu, N 232

Tukiainen, E 1444

Tummino, PJ 1717

Tunici, P 303

Turley, H 742, 1749

Tveit, KM 1282

Tye, L 1543

Tzardi, M 465

Uchida, K 1648

Uchihashi, Y 1425

Uehara, Y 145, 1220

Ueno, H 908

Ueno, K 1374

Ullah, R 410

Ulmer, H 1202

Umana, P 1758

Unoki, M 98

Urasaki, Y 983

Urashima, M 1957

Ushijima, K 967

Utley, M 541

Uzawa, K 684

Vaarwater, J 813

Vaccarella, S 865

Vacherot, F 951

Vainer, B 530

Vaissiere, N 232

Valero, V 1813

Validire, P 473

Valle, JW 621

Vallera, DA 1114

van Agthoven, T 1824

van Bodegraven, AA 1671

Van Calster, B 244

Van Cutsem, E 1033

van de Nieuwenhof, HP 27

van de Wiel, MA 707

van den Bent, M 1995

Van Den Berg, DJ 1805

van den Eertwegh, AJM 1224

van den Ouweland, A 2048

van der Poel, H 1505

van der Velden, PA 312

van der Veldt, AAM 1224

van der Woude, CJ 1671

van der Zee, AGJ 149

van Herpen, CML 219

van IJcken, WFJ 1824

van Kemenade, FJ 202

Van Laere, SJ 628, 1028

van Leeuwen, FE 1505

van Oijen, MGH 1274

van Os, TAM 1456

van Rijn, AF 1274

van Rossum, LGM 1274

Van Segbroeck, S 1130, 2066

Van Seuningen, I 637

Vandenput, I 244

Vara, D 940

Varey, AHR 1183

Varon-Mateeva, R 2048

Vasudev, NS 1175

Vatten, LJ 534

Végran, F 1357

Veiga, FJG 1248

Veldscholte, J 1824

Venoux, C 673

Verbeek, ALM 1274

Vergote, I 244

Verhoef, S 2048

Vermeulen, E 1505

Vermeulen, K 628

Vermeulen, PB 1028

Vermorken, JB 628

Versmold, B 1456, 2048

Verspaget, HW 765

Verwaal, VJ 1505

Vessella, RL 263

Vidnovic, N 1606

Viel, A 2048

Viel, E 734

Vierkant, RA 1461, 1805

Viktorsson, K 816

Villanova, G 232

Vincenzi, B 715

Visintin, I 335

Vitonis, AF 1805

Vizzielli, G 1066

Vleggaar, FP 1671

Voigt, W 1241

Voirin, N 673

Vonen, B 1282

Vorgias, G 1059

Vredenburgh, JJ 1986

Wada, T 1425

Wagner, AD 1846

Wagner, CR 71

Wai, DH 80

Wakamatsu, T 1676

Wakeley, K 2048

Walker, C 124

Wallach, T 1481

Walsh, CP 441

Walters, SJ 561, 568

Wang, C 1513

Wang, E 1543

Wang, L 432

Wang, R 465

Wang, S 774

Wang, X 1461, 2048

Wang, Z 327, 1513

Ward, DG 295

Ward, T 55

Wardley, AM 582

Wasan, HS 621, 1162

Watanabe, J 1676

Watanabe, M 116

Watson, AJ 550

Waugh, DJJ 1620

Webb, PM 1805

Weber, AA 2038

Weber, H 457

Weerasooriya, N 2048

Wei, W 295, 1393

Wein, A 1846

Weir, A 1995

Weiswald, L-B 473

Weitz, J 457

Welch, K 238, 2062

Werner, M 1513

Wethal, T 575

Wever, EM 1833

Wheatley, ER 1183

Whitaker, NJ 1345, 1351

White, NMA 1107

Whiteway, J 1839

Whitson, BA 424

Whittemore, AS 1461, 1805

Whitworth, J 403

Widschwendter, M 160

Wiedenmann, B 1574

Wiesinger, H 1241

Wijnen, JT 2048

Wikström, I 871

Wilander, E 871

Wilding, JL 1758

Wilkens, LR 185, 1805

Wilkinson, SJ 498

Wilkowski, R 1853

Willems, L 1497

Willett, WC 916

Williams, KJ 1290

Willis, W 774

Wilson, RH 1620

Wilson, S 250

Winterhoff, B 1699

Wise, M 924

Wishart, GC 875, 1338, 1522

Wlazlinski, A 1410

Wohlfahrt, J 530

Wolk, A 1932

Wolpin, BM 916

Wong, CYP 691

Wong, H 124

Wong, R 1162

Woolgar, JA 139

Workman, P 1860

Wouters, A 628

Wright, KE 658

Wu, AH 1805

Wu, K 916

Wu, MH 1635

Wyld, L 1692

Xenidis, N 589

Xia, L 38

Xie, X 1798

Xie, Y 342

Xu, M 303

Xu, Q 303

Yagita, H 1709

Yamaguchi, T 967

Yamamoto, J 908

Yamamoto, N 225

Yamasaki, M 1298

Yamashita, T 1425

Yamato, I 1709

Yamatoji, M 684

Yamazaki, K 598, 1031

Yamori, T 350

Yan, M 1168, 1806

Yanaihara, N 1957

Yang, D 774

Yang, GR 1699

Yannoukakos, D 32, 2048

Yano, T 145, 1220

Yao, KM 1433

Yashiro, M 1100, 1365

Yassin, Y 2048

Ye, F 1798

Ye, Y 1345, 1351

Yee, D 71

Yeh, W-C 1555

Yokota, N 2023

Yokoyama, A 1549

Yokoyama, M 1884

Yokozaki, H 1365

Yonei, T 1537

Yonemori, K 1529

Yoo, C 1658

Yoo, K-Y 526

Yoon, H-S 822

Yoshida, T 492

Yoshioka, T 1972

Yossepowitch, O 2057

You, S-L 174

Young, J 342

Yousef, GM 1107

Ythier, A 1051

Yu, EY 263

Yu, J 691, 699

Yu, JS 303

Yu, W-Y 342

Yu, Y 327

Yuan, X 303

Yudina, Y 1596

Yun, SH 2015

Yuuki, K 2005

Zacharakis, E 19

Zaremba, T 256

Zeng, X 71

Zerrouki, S 1417

Zhang, H 71, 935

Zhang, SW 1635

Zhang, WY 1635

Zhang, YP 1635

Zhao, H 897

Zhao, R 1635

Zhao, YZ 327

Zhong, W 2015

Zhu, J 327

Zhu, L 1635

Ziegler, RG 178

Zikan, M 2048

Zinkewich-Peotti, K 1807

Ziogas, A 1805

Zismanov, V 1402

Zlobec, I 1382

Zuhlke, KA 2043

